# Long Non-coding RNAs: Mechanisms, Experimental, and Computational Approaches in Identification, Characterization, and Their Biomarker Potential in Cancer

**DOI:** 10.3389/fgene.2021.649619

**Published:** 2021-07-01

**Authors:** Anshika Chowdhary, Venkata Satagopam, Reinhard Schneider

**Affiliations:** Luxembourg Centre for Systems Biomedicine, University of Luxembourg, Esch-sur-Alzette, Luxembourg

**Keywords:** lncRNAs, cancer, biomarker, mechanisms, methods

## Abstract

Long non-coding RNAs are diverse class of non-coding RNA molecules >200 base pairs of length having various functions like gene regulation, dosage compensation, epigenetic regulation. Dysregulation and genomic variations of several lncRNAs have been implicated in several diseases. Their tissue and developmental specific expression are contributing factors for them to be viable indicators of physiological states of the cells. Here we present an comprehensive review the molecular mechanisms and functions, state of the art experimental and computational pipelines and challenges involved in the identification and functional annotation of lncRNAs and their prospects as biomarkers. We also illustrate the application of co-expression networks on the TCGA-LIHC dataset for putative functional predictions of lncRNAs having a therapeutic potential in Hepatocellular carcinoma (HCC).

## Introduction

Advancement in Next Generation Sequencing (NGS) technologies and genome wide analysis of gene expression have revealed at least 80% of the human genome is active (Palazzo and Lee, 2015). However, only up to 1.5% of the genome is translated to protein which implicate RNAs to have more diverse roles than an intermediate component as templates in the genetic flow of information from gene to protein. They are categorized into mRNAs which are translated into proteins and non-coding RNAs (ncRNAs) which have little or no coding potential but are involved in transcriptional regulatory mechanisms.

The evolutionary development of an organism is associated with the increase in complexity of the regulatory potential of these ncRNAs which constitute the majority of the transcriptome. Non-coding RNAs are further categorised as short ncRNAs which include microRNAs (miRNAs), small RNA (sRNA), piwi-interacting RNAs (piRNAs), siRNAs, and long non-coding RNAs (lncRNAs) consisting long intergenic non-coding RNAs (lincRNAs), circular RNAs (circRNAs), and competitive endogenous RNAs (CeRNAs) (Hombach and Kretz, 2016). These RNAs have known to have functions involved in cellular functions like mRNA translation, alternative splicing events, RNA editing and also regulatory mechanisms like RNA silencing involving miRNA and mRNA interference via siRNA (Mattick and Makunin, 2006). LncRNAs have emerged as a latest class of RNA molecules which are more diverse than short ncRNAs having complex gene regulatory functions in the cells. In this article we present and review the various biological characteristics and mechanisms of lncRNAs in transcriptional regulation and the latest development in experimental and computational methods for their identification, annotation and putative function prediction.

There are more than 30,000 lncRNAs in humans available in the GENCODE (Harrow et al., 2012), and more and more new lncRNAs are being discovered overtime. Long non-coding RNAs are typically longer than 200 nucleotides of length and sometimes have similar features to that of protein-coding genes, such as a 5' cap, exons and poly A tail and are spliced post-transcriptionally, but don't possess functional open reading frames and cannot be translated to functional proteins. (Fang and Fullwood, 2016). Their varied molecular properties enable them to function in various methods regulating gene expression at various stages of cellular development (Hanahan and Weinberg, 2000).

LncRNAs are also not stable in comparison to mRNAs, localized mainly across the nucleus and cytoplasm and also not conserved across species, transcribed mostly by RNA polymerase II and exhibit tissue specific expression. However, high conservation patterns have been observed in the exonic regions and promoters regions of the lncRNA. Recently, it has been discovered that some lncRNAs can in fact translate to small peptide chains which could have significant biological functions (Hubé and Francastel, 2018; Li and Liu, 2019).

One way to classify lncRNAs is based on the genomic locations from where they are transcribed relative to protein coding genomic regions: (1) lincRNAs: long intergenic non-coding RNAs which are transcribed from the intergenic regions between the protein coding genes; (2) Sense lncRNAs: transcribed from the sense strand of the protein coding genes and may overlap with a part or the entire sequence of a protein coding gene; (3) Antisense lncRNAs: transcribed from the antisense strand of the protein coding genes which may overlap of exons, only from the intronic region and overlapping the entire gene in the antisense strand. (Ma et al., 2013); (4) Intronic lncRNAs: transcribed from the intronic regions between the exomes of a gene. (5) Bidirectional lncRNAs: transcribed from both sense and antisense directions of TSS (Hanahan and Weinberg, 2000; He et al., 2014, 2017).

## Functions and Mechanisms of Long Non-Coding RNAs

**Figure 1:**
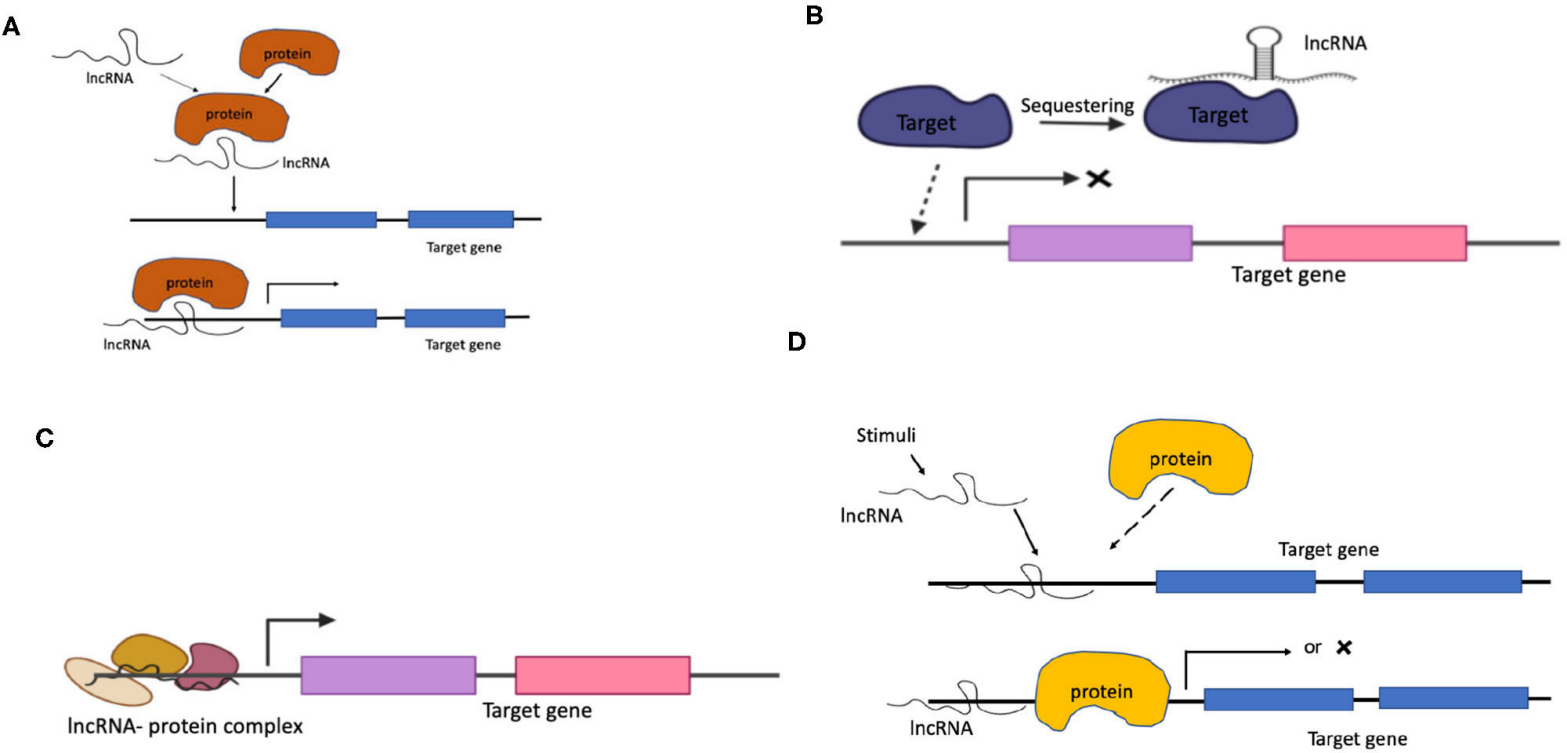
Mechanisms of lncRNA. **(A)** Signals, **(B)** Guides, **(C)** Decoys, and **(D)** Scaffolds.

The elucidation of the mechanisms of long non-coding RNAs is mostly based on empirical evidence of the subcellular localization, developmental stage of the cell and tissue specific expression. The function of lncRNAs can be stratified into four types of molecular mechanisms described and illustrated (Figure 1; Zanella, 2021) below.

###  Signals

Transcriptional regulation aided by lncRNA where they function as signals are brought by various factors like developmental stages, organismal stress, re-programming of cells and state of the cell at a particular space and time in response to the environment and their expression could be a phenotypical indicator of these states (Wang and Chang, 2011). A prominent example is the chromatin regulation for dosage compensation in females in X-chromosome inactivation (XCI) (Engreitz et al., 2013; Wasko et al., 2019). The mechanism includes expression of XIST lncRNA from one of the X chromosome which coats itself leading to its silencing, which is also aided by the accumulation of the lncRNA Jpx. The antisense transcript of XIST, TSIX represses the activity of XIST in the other chromosome rendering it to be active (Starmer and Magnuson, 2009; Wang and Chang, 2011; Carmona et al., 2018). Another example of epigenetic re-programming that takes place in plants mediated by lncRNAs is to switch between vegetative to reproductive state. In *Arabidopsis thaliana* with the decrease in temperature for an extended period of time during winter COOLAIR is expressed and accumulated in large amounts which represses the expression of the FLOWERING LOCUS C (FLC). This is gene mediated by the PRC2 complex which when expressed normally in winter stops flowering in the plant. So, gradually upon the approach of spring and warmer temperatures COOLAIR enables vernalization of plants (Swiezewski et al., 2009; Tian et al., 2010; Heo and Sung, 2011; Wang and Chang, 2011).

###  Guides

As guides lncRNAs bind to proteins and direct them to specific sites, also leading to expression or silencing of the target genomic regions. This essentially involves recruitment of chromatin modifying enzymes which alter the chromatin state with the formation of complex structures with RNA-RNA, RNA-DNA, RNA-DNA-effector proteins. For instance, XIST transcription has also known to be induced by recruiting the Polycomb Repressive Complex 2 (PRC2) by RepA RNA. Additionally XIST also interacts with a matrix protein hnRNP U for its accumulation at the chromosome (Wang and Chang, 2011). Some other examples of lncRNAs acting as signals and guides include COLDAIR, HOTTIP, HOTAIR, ROR and some PRC2-bound RNAs (Rinn et al., 2007; Loewer et al., 2010; Wang et al., 2011; Kim et al., 2017).

###  Decoys

LncRNAs can also regulate transcription by acting as endogenous target mimics (eTMs) where they bind to intermediary regulatory proteins, RNA, DNA molecules and sequester them away from their respective target site. These otherwise known as competitive endogenous RNA (ceRNA) act as *sponges* generating a “sponge effect” by base pairing with target molecules which include transcription factors, miRNAs, chromatin modifiers (Wang and Chang, 2011) among others at their active sites and render them to be unavailable for interaction for their target molecules. An example of such activity is that of the lncRNA transcribed at the minor promoter of the DHFR gene which pairs and forms a complex with the DNA at the promoter region of the same gene. The complex inhibits formation of the preintiation complex and also interacts with transcription factor IIB (TFIIB) which was also further confirmed by siRNA knockdown of the lncRNA (Martianov et al., 2007; Wang and Chang, 2011). MALAT1 (Tripathi et al., 2010), TERRA (Redon et al., 2010), Gas5 (Kino et al., 2010) are also examples that exhibit the 'sponge'/sequestering mechanism. ceRNA mechanism has been extensively studied with several computational algorithms and repositories also being developed in order to identify and store potential and experimentally verified targets of lncRNA (listed in Table 1). However, verification of their mechanism have to be contended with transcriptional levels of miRNA and lncRNA to be sufficient enough for them to function as competitive endogenous RNAs (Denzler et al., 2014, 2016; Zhang et al., 2019).

**Table 1:** Databases and computational pipelines predicting lncRNAs functioning as ceRNAs.

**Databases/Computational pipeline**	**Description**	**References**
DIANA-LncBase v3	Database dedicated to cataloging miRNA and lncRNA interactions, includes ceRNABase	Karagkouni et al., 2020
StarBase v2.0	RNA-RNA and protein-RNA interactions from CLIP-Seq experiments predicting ceRNA function	Li et al., 2014
spongeScan	predicts miRNA target sites in lncRNAs	Furió-Tarí et al., 2016
lnCeDB	stores lncRNAs acting as ceRNAs with targets from StarBase and TargetScan Grimson et al., 2007	Das et al., 2014
LncCeRBase	lncRNA-miRNA-mRNA interactions collected from literature	Pian et al., 2018
Linc2GO	predicts linc RNAs functions using miRNA and mRNA interactions based ceRNA hypothesis	Liu et al., 2013

###  Scaffolds

LncRNAs serve as structural supports where other effector proteins and DNA/RNA molecules bind to form a functional complex and are then directed to appropriate localization of the complex for its function. Gene repression by HOTAIR forming a complex with the polycomb complex PRC2 for methylation at H3K27 (Rinn et al., 2007; Wang and Chang, 2011) and also forming a complex with LSD1, CoREST and REST (Wang and Chang, 2011) exhibits this mechanism. TERC also assembles the telomerase complex and mediates reverse transcriptase activity by binding with telomere targeting proteins (Balas and Johnson, 2018). The lncRNAs ANRIL (Yap et al., 2010; Kotake et al., 2011), SRP(Signal Recognition component), LINP1(LncRNA In Nonhomologous End Joining Pathway 1) (Sakthianandeswaren et al., 2016) are also found to have similar mechanisms.

## Identification and Annotation

###  Experimental Approaches

Widely used experimental approaches to identify and annotate lncRNAs include Microarray, RNAseq, SAGE, CAGE among others with customized adaptations to identify and annotate lncRNAs based on their molecular characteristics as described in the following sections and listed in Table 2.

**Table 2:** Experimental approaches in lncRNA profiling.

**Experimental approaches**	**Features**
RNA-seq	Identifies on novel lncRNA transcripts
Microarray	Reannotations of existing microarrays
	Arrays specifically designed for lncRNAs
Tiling arrays	Ability to profile transciptome for specific regions(whole) in the genome.
SAGE	Accurate quantification and novel transcript identification
CAGE	Identification of transcription start points
PARE, degradome-seq	Used in RNA degradome analysis
GRO-seq	Measures nascent RNA regulating gene transcription
RIP, CLIP	LncRNA-protein interaction identification
TIF-seq	Identification of isoforms of lncRNA
Selective 2'-hydroxyl acylation by primer extension (SHAPE)	LncRNA structure prediction
PARS	LncRNA structure prediction *in vitro*
FragSeq	Transcript structure prediction from RNA fragments
nextPARS	Adaptation of PARS to Illumina technology

###  Adaptations in Microarray Technology

Probesets in conventional microarray platforms do not have lncRNAs annotations and not suitable for identifying and measuring lncRNA levels. Some of the mRNAs from these previous microarrays that have been correctly identified as lncRNAs have been re-annotated and their expression levels have been re-analyzed accordingly (Michelhaugh et al., 2011; Ma et al., 2012). ArrayStar Human LncRNA microarrays (V4.0) has been designed to profile both lncRNA and mRNA on the same array with 40,173 lncRNAs with 7,506 gold standard lncRNAs, 20,730 mRNAs among 60,903 distinct probes (Shi and Shang, 2016). As the expression of lncRNAs indicates the relative physiological state of a cell, differential expression between samples at different conditions can provide us information to understand the regulatory lncRNAs at these conditions. (Zhang et al., 2017) identified novel circulating lncRNAs: TINCR, CCAT2, AOC4P, BANCR, and LINC00857 which are differentially expressed in gastric cancer patients and be detected from the plasma of patients and hence function as biomarkers. Similarly, it was found that the lncRNA ENST00000551152 was upregulated and the lncRNA TCO.NS_00001368 was downregulated in cervical cancer cell lines (Huang et al., 2018) in a study by Huang et. al using Agilent DNA microarray. Whole-genome tiling arrays are used for the sequenced regions which are not annotated for lncRNA isolation and identification. (Lund et al., 2014) used this in their experimental design where they used tiled probes from chr8: 127,640,000–129,120,000 at locus 8q24 to analyze prostate tissue from prostate cancer patients.

###  RNA-Seq Technologies

RNA-seq is the most prevalent technique used to identify and annotate novel long non-coding transcripts that are less abundant including the isoforms of lncRNAs. RNA-seq offers a broad spectrum of transcript identification with novel transcripts detection and *de novo* assembly as probes are not required in order to hybridize and capture transcripts from samples. Modifications in the RNA-seq pipeline facilitate identification of specific type of lncRNAs, for instance strand-specific RNA-seq allows labeling of *origin of strand* information on the transcripts which allows sense/antisense lncRNA segregation and identification (Mills et al., 2013; Liu et al., 2019).

Wang et al. identified 2895 novel lncRNA in endometrial tissue of pigs; of which 301 were differentially expressed and functionally annotated to be involved in several biological pathways including immune system process and other cellular process of which TCONS_01729386 and TCONS_01325501 have a major functions in embryo pre-implantation (Liu et al., 2017). Functional attributes of lncRNA are validated with qRT-PCR experimental pipelines in which siRNA, GAPmers are designed to knockdown the lncRNA and the resulting change in gene expression is analyzed to identify its effector genes/molecules. However, in order for *in vitro* studies to correlate with vivo studies several contributing factors involved in the knockdown of lncRNA and its effect on resulting varying gene expression need to be considered. Features of the lncRNA to consider while design of the knockdown strategy is the sub-cellular localization of the lncRNA, along with the developmental stage of the cells. Lennox et al. were able to decipher that nuclear lncRNAs were knocked down at higher levels using antisense strands and cytoplasmic lncRNAs were better knocked down using RNAi (Lennox and Behlke, 2016). In a recent study by Nicola Amod et al. a MALAT1-targeting 16mer LNA gapmeR g#5 showed significant anti-tumor activity in humanized murine model. Inference from transcriptome analysis showed proteasome expression was repressed by g#5 and was instead enriched increased *in vivo* in MALAT1 murine model patients (Amodio et al., 2018). RNA CaptureSeq (Mercer et al., 2011), another derivative of RNA-seq involves tiling arrays prepared for specific target regions of the genome. cDNAs against these regions are hybridized and sequenced. This method supports the identification of novel unannotated lncRNAs along with high fold coverage.

###  SAGE, CAGE

Serial Analysis of Gene Expression (SAGE) (Velculescu et al., 1995) and Cap Analysis of Gene Expression (CAGE) are based on short sequences tags which are complementary to a given RNA of interest (Kashi et al., 2016). In SAGE these cDNA tags are biotinylated, captured on streptavidin beads (Wang and Chekanova, 2019). They are further ligated and later PCR amplified followed by concatenation and sequencing by mapping to reference genes. This method like RNA-seq facilitates discovery to novel transcripts and enables accurate measurement of expression levels of lncRNAs but has a drawback of small cDNA sequences mapping to multiple genes in the reference genome. Gibb et al. analyzed 272 SAGE libraries normal(26) and cancer(19) tissues from human which elucidated the tissue specific and aberrant expression lncRNAs in cancer tissues implicating them in disease development (Gibb et al., 2011). In a study by Jia et al. (2018) SAGE datasets of OPL(Oral premalignant lesions) from GEO were analyzed to identify 10 differentially expressed lncRNAs among with the lncRNA NEAT1 was the highly expressed in OPL. NEAT1 has been also implicated in lung cancer metastasis and hepatocellular carcinoma (Dong et al., 2018).

Cap analysis gene expression (CAGE), was a development upon SAGE to over come its drawbacks where cDNA tags can be generated from the 5′ end of the RNA of interest. The cap structure of the transcripts are biotinylated in the CAP-trapper method followed by cDNA tag generation, cleaving by restriction enzymes, PCR, ligation and cloning of tags and mapping to reference genome (Shiraki et al., 2003). CAGE allows the expression analyzes at promoter regions but is restricted only to capped RNAs. CAGE method has better throughput with the use of sequence tags and is also cheap in comparison to cDNA library (Shiraki et al., 2003). Hon et al. (2017) collated 27,919 human lncRNAs from 1,829 datasets from CAGE and other methods in the FANTOM5 project. HeliScopeCAGE (Kanamori-Katayama et al., 2011) nanoCAGE (Poulain et al., 2017) CAGEscan (Bertin et al., 2017), DeepCAGE (Valen et al., 2009) are also protocols based on the CAGE technology for profiling the mammalian transcriptome.

###  Other Approaches

Parallel analysis of RNA-ends (PARE) (German et al., 2008), genome-wide mapping of uncapped transcripts (GMUCT) (Gregory et al., 2008), degradome-seq are among other techniques developed to map transcripts that are not stable and get degraded i.e., they act as templates for other non-coding RNAs like miRNA. RNA-seq measures transcripts at equilibrium conditions where as on the other hand Gro-seq (Global run-on sequencing) is able to sequence nascent RNA. This has revealed genome wide view of the transcripts by measuring half life of transcripts at various time points. RNA-seq and GRO-seq analyzes have revealed that divergent transcription occurs at the promoter regions of protein-coding genes (Kashi et al., 2016). 5'-bromo-uridine immunoprecipitation chase—deep sequencing analysis (bric-seq) method involves labeling of transcripts with 5'-bromo-uridine (BrU) which are isolated at sequential time intervals and recovered by immunopurification followed by RT-qPCR (Tani et al., 2012; Kashi et al., 2016). TIF-seq, an approach developed by Pelechano et al. (2013), jointly sequences both 5' and 3' ends of RNA molecules enabling characterization isoform heterogeneity of RNA molecules.

Other than perturbation by silencing of lncRNAs by RNA interference as mentioned in above section, functional characterization of lncRNA also involves methods like RNA centric purification methods when the RNA is pulled down exogenously based on *in vitro* affinity capture methods or endogenously under native or ultraviolet (UV) cross-linking conditions (Cipriano and Ballarino, 2018). On the other hand protein centric purification involves immunoprecipitation of lncRNAs and their target proteins with specific antibodies. RNA immunoprecipitation (RIP) is used to functionally characterize the lncRNA by purifying RNAs associated with target proteins. Cross-linking immunoprecipitation (CLIP), combination of CLIP with high-throughput sequencing (HITS-CLIP or CLIP-seq) and Photo Activatable Ribonucleotide-enhanced (PAR-CLIP) (Spitzer et al., 2014) been developed to analyze interactions of RNA binding proteins but these methods carry disadvantages like loss of cDNAs and de-crosslinking along with being expensive (Barra and Leucci, 2017). Chromatin isolation by RNA purification (ChIRP) has been used to identify lncRNAs and their interactions with chromatin during gene regulation (Chu et al., 2011; Kashi et al., 2016). Further more, techniques have been developed to probe the RNA structures, such as Selective 2' -hydroxyl acylation by primer extension (SHAPE) [67], parallel analysis of RNA structure (PARS) (Kertesz et al., 2010) and FragSeq (Underwood et al., 2010) which can provide an extensive evidence on mode of action and interactions with other regulatory molecules (Guo et al., 2016). More recently, Saus et al. described nextPARS an adaptation to PARS technique on the Illumina's sequencing technology where parallel execution of highly specific enzymatic digestion of single an double stranded genomic regions make the “capable of tagging both all the bases in single and double-stranded conformation at a genome-wide scale” making it cost effective with better throughput (Saus et al., 2018). CRISPRlnc, containing manually curated and validated 2184 CRISPR/Cas9 sgRNAs for 335 lncRNAs from different species, (Chen et al., 2019) was developed by Chen et al. which would further help design CRISPR/Cas9 experiments to investigate lncRNAs functions.

###  Computational Approaches

Novel Computational tools and pipelines are quintessential in combination with novel experimental techniques to identify putative transcripts as lncRNAs and further elucidate their functional roles involving interactions with other DNA, RNA and proteins. Computational pipelines to process NGS data are modified for the annotation of putative lncRNAs from novel transcripts. For the genome wide identification of lncRNA transcripts from data sets generated by the most widely RNA-seq techniques for novel lncRNA identification typically involves the following steps: alignment of reads from the experiment to the target regions in reference genome. This is followed by transcripts assembly and isoform identification and scoring the transcripts for protein coding potential (Coding Potential Calculator) (Jalali et al., 2015) and also include attributes like presence of open reading frames, poly-A tails and exonic regions and strand information into consideration. Standard programs like HISAT2, (Trapnell et al., 2009), STAR (Dobin and Gingeras, 2015) are used for mapping and StringTie (Ghosh and Chan, 2016), Scripture (Schoenbeck, 2016) for assembly. After transcripts of length >200 bp are filtered out, other types of transcripts such as tRNA, rRNA, snoRNA, miRNA, siRNA etc are searched in different databases and removed. Following this, based on their homology scores using programs like BLAST, BLAT the candidate lncRNAs are annotated with information from lncRNA databases. Sequence alignment and similarity search methods such as BLASTX and HMMER3 (Eddy, 2009) search against data repositories like UniProt, PDB and filter RNA transcripts which have similar homologous domains and can be translated to proteins (Gish and States, 1993; Eddy, 2011; Jalali et al., 2015). On comparing the performance of various alignment methods (Zheng et al., 2019) Kallisto or Salmon in combination with full transcriptome annotation performed best for lncRNA detection on both un-stranded and stranded RNA-Seq datasets.

ORF is also among the features which help categorization of novel transcripts as lncRNAs; for example ORF length predicted by EMBOSS tools (Itaya et al., 2013) (getORF). ORFs of length greater than 100 codons categorised as mRNAs are filtered out as coding transcripts but it is not a definite threshold with certain exceptions like XIST, H19 among others which having ORFs longer than 100 amino acids (Dinger et al., 2008; Jalali et al., 2015).

Another approach is use of machine learning based tools developed on SVM, logistic regression models use sequence features to compute the protein coding potential which predict the transcript to be a lncRNA/mRNA. ORF, conservation of the exonic regions of the transcript, nucleotide composition, sequence motif and codon usage are inclusive feature vectors from the transcript sequences to train the models. In order to compute transcript's coding potential two methods have been developed CPC (Coding Potential Calculator) (Altschul et al., 1997; Kong et al., 2007; Ma et al., 2012) based on SVM models with sequence features and the comparative genomics features and ii) A later faster version CPC2 that can be for novel transcripts of organism which have improper genome assembly and poorly annotated (Kang et al., 2017). CONC (for coding and non coding) (Liu et al., 2006) also trains SVM models based on a comprehensive set of RNA features like the peptide length and composition, secondary structure, compositional entropy among others to classify transcripts as lncRNAs and mRNAs. Lu et al. have further integrated quantitative properties like a GC content, conservation patterns, level of expression which is lower of lncRNAs in comparision to mRNAs to predict lncRNAs in *C. elegans* in their machine learning model (Lu et al., 2011; Ma et al., 2012). The pipeline employed by Sun et al. lncRScan-SVM (Sun et al., 2015), which after a standard processing of RNASeq transcripts identifies transcripts as lncRNAs by a SVM model trained on GTF positive and negative samples. iSeeRNA is also a similar tool that identifies putative lincRNAs by on SVM based classifier (Sun et al., 2013). COME, a coding potential calculator, developed by Hu et al. (2017) integrated multiple features from both sequences and experiments like poly(A) enrichment, methylation taken from RNA-seq data sets had more accuracy over transcripts of different lengths. In the COME method, an index for the whole genome splitting it into bins of 100-nucleotide(nt) on which the feature vectors were generated and subsequently a balanced random forest (BRF) was trained.

Attempts to functionally characterize novel lncRNAs by computational methods have been challenging. In the case of protein-coding genes a putative function is assigned to transcripts based on their similarity with already characterized proteins (de Hoon et al., 2015); as they have highly conserved regions across species which is not the same with lncRNAs. Their tissue specificity and low abundance along with varied mechanisms involved with various other biological molecules further add to the complexity of modeling their functionality *in-silico*.

#### Co-expression Evidence Analysis and Network Inference

Data analysis of microarrays and tiling experiments include identification of differential expressed transcripts followed by network analysis based on co-expression patterns. To infer the putative function of a lncRNA 'guilt by association' algorithm has been developed based on the co-expression patterns of lncRNA and protein coding genes (PCGS) which suggest their functional relatedness and regulatory relationships. The tissue and condition specific expression, subcellular localization are distinctive attributes of lncRNA expression which are combined with differential expression to infer putative functions and target proteins interactions of the lncRNA and their role in disease development (Li et al., 2016; Gao et al., 2019a). The correlation scores between expression profiles of lncRNAs and PCGs at a given condition/tissue/time series are calculated which represents a network by a transformed correlation-adjacency matrix. From these networks, clusters of co-expressed lncRNAs and mRNAs are identified. The functional regulation of lncRNAs are annotated based on the functional enrichment of the PCGs in the clusters with which it is co-expressed.

Co-lncRNA is one such tool/database developed by Wu et al. (2016) where they were able to analyze lncRNA-mRNA co-expression patterns, consistent with previous established related lncRNA-mRNAs like HOTAIR, BRCA2, MMP9 and MMP11 and also novel lncRNA RP11-118E18 validated by TANRIC. Such network based clustering approaches have also been further extended to include other non-coding RNAs and regulatory proteins like miRNAs to predict more specific mechanisms like cis-regulatory relationships where whole transcriptomic data is analyzed (Signal et al., 2016).

Several studies have been done to understand the pathogenesis of complex diseases from available data of lncRNA and their interacting proteins (Sumathipala et al., 2019). The approaches consist of Machine learning (ML) based models trained over expression profiles to extract patterns from which lncRNA functionality and disease associations are predicted, random walk based models on networks representing the similar expression patterns or a combination of both. (Chen and Yan, 2013) included disease information into identify lncRNA disease associations from lncRNA expression levels by developing a semi-supervised learning model Laplacian Regularized Least Squares for LncRNA Disease Association (LRLSLDA).

Chen et al. further developed novel lncRNA functional similarity calculation models (LNCSIM) by associating the semantic similarity between lncRNA and disease groups (Chen and Yan, 2013; Chen, 2015a,b). Guo et al. (2019) developed LDASR to identify lncRNA-disease associations where Guassian profile similarities and neural network for dimensional reduction and finally rotating forests were used to predict disease associations (Guo et al., 2019). DislncRF also uses random forest models trained over lncRNA-disease associated protein coding genes in order to score the association of lncRNA for a particular disease (Pan et al., 2019). Liao et al. developed a method called GrwLDA which is based on global network random walk model in order to predict lncRNA and their associated diseases (Gu et al., 2017). Xuan et al. (2019) also recently proposed a tool graph convolutional network and convolutional neural network (GCNLDA) to explore network and come up with lncRNA-disease candidate pairs. Bipartite Network inference (LPBNI), a computational pipeline developed by Ge et al. (2016) used two-step propagation in the bipartite network to rank target proteins for lncRNAs; BPLLDA developed to predict lncRNA-disease links from a network of heterogenous lncRNAs and associated diseases based on their node interaction paths (Xiao et al., 2018). TPGLDA also had been developed to predict lncRNA-disease from lncRNA-disease-gene tripartite graph constructed base on was developed by Ding et al. (2018) where they could predict lncRNAs like GAS5, UCA1, implicated in lung, hepatocellular, ovarian cancer (Ding et al., 2018). The above mentioned tools are all based on network propagation and inference. Recently, a similar network diffusion algorithm called LION was developed to infer key candidate lncRNAs (Sumathipala et al., 2019) by Sumathipala et al. with better prediction results for cardiovascular diseases and cancer. Another recent approach IDHI-MIRW by Integrating Diverse Heterogeneous Information(IDHI) with positive pointwise Mutual Information and Random Walk(MIRW) was also proposed by Fan et al. (2019) which integrates lncRNA-miRNA/protein and expression profiles along with disease ontology information.

#### Conservation and Structure Prediction

Although the conservation scores of lncRNA molecules are lower than mRNA, when used within awareness of biological context including information about potential interactions with other RNA, DNA, proteins, can decipher evidences to categorise novel transcripts to lncRNAs. Algorithms like BLAST (Altschul et al., 1990), ClustalW (Thompson et al., 2002), MAFFT (Katoh et al., 2009), ConSurf (Glaser et al., 2003), MUSCLE (Edgar, 2004) among others perform multiple sequence alignment. Furthermore tools like RNAz 2.0 (Gruber et al., 2010), Evofold (Pedersen et al., 2006) can predict conserved RNA structures from multiple sequence alignment. RNAstructure (Reuter and Mathews, 2010), GTFold (Swenson et al., 2012), CentroidFold (Sato et al., 2009), RNAfold (Denman, 1993), Mfold (Zuker, 2003), CentroidHomfold-LAST (Hamada et al., 2011), and Seqfold (Ouyang et al., 2013), FARNA (Alam et al., 2017), iFoldRNA (Sharma et al., 2008) are among the tools to predict RNA secondary and tertiary structures, respectively from primary sequence. The RNA-RNA interaction prediction methods mainly employ alignment algorithms, comparative (homology) methods and *in silico* energy calculations (Umu and Gardner, 2017). Minimum Free Energy based methods are based on computation of the minimum free energy of the RNA-RNA molecules taking the inter- and/or intra molecular base-pairing into account. On the other hand, as perceivable, alignment and homology based methods include algorithms using tools for multiple sequence alignment and seed match-extension.

IntaRNA (Mann et al., 2017), RNAhybrid (Krüger and Rehmsmeier, 2006), Pairfold (Andronescu et al., 2003), RNAplex (Tafer and Hofacker, 2008), RIsearch (Wenzel et al., 2012), RIblast (Fukunaga and Hamada, 2017), Bindigo (Hodas and Aalberts, 2004), and GUUGle (Gerlach and Giegerich, 2006) are some examples of tools used to predict RNA-RNA interactions. These are also integrated in pipelines to predict lncRNA-RNA interactions in humans. For instance, (Terai et al., 2016), developed a pipeline using RACCESS (Kiryu et al., 2011) to extract accessible regions from RNA molecules followed by masking tandem repeats using TanTan (Frith, 2011) and finding seed match using LAST and then calculate the interaction energy between two RNA molecules using IntaRNA and finally predict the joint secondary structure (RactIP) (Kato et al., 2010) to predict lncRNA-mRNA interactions (Szcześniak and Makałowska, 2016) proposed a similarity based method to predict RNA-RNA interactions using LAST (Kiełbasa et al., 2011), miRanda (Betel et al., 2010) tools in some pipelines. Similarly, RNA-protein interactions are also be predicted from sequence based methods which use physiochemical properties of amino/nucleic acids in tools like lncPRO (Lu et al., 2013) and catRAPID (Bellucci et al., 2011). Along with these sequence features, secondary structures of RNA are incl in tools like RPI-Pred (Suresh et al., 2015). PARIS (Lu et al., 2016), SPLASH (Aw et al., 2016), LIGR-seq (Sharma et al., 2016), and MARIO (Nguyen et al., 2016) to identify RNA-RNA interactions based on proximity ligation *in vivo* (Fukunaga and Hamada, 2017).

## LncRNA Databases

The publicly available datasets from RNA-seq and microarray experiments have led to rapid increase of annotated lncRNAs with dedicated databases for lncRNA and their molecular and disease associations. Many pipelines and tools have been benchmarked from the data available from these knowledge bases. NONCODEv5, the largest database for noncoding RNAs (majorly lncRNAs) contains 548,640 lncRNA transcripts from several model organisms (Fang et al., 2018), of which 96,308 lncRNA genes are from humans. The data has been curated from published literature and annotated with information from public resources like RefSeq, Ensembl, GenBank, lncRNAdb, lncipedia. The FANTOM (Functional ANnoTation Of the Mammalian genome) consortium led by RIKEN has systematically investigated and annotated about 27,919 human lncRNA genes across 1829 samples in the FANTOM database (FANTOM5) (Abugessaisa et al., 2017). Some of the databases provide experimentally validated and/or computationally predicted interactions of lncRNAs with other RNA and proteins. Analysis of data from RNA-seq and microarray experiments on disease cell lines have also helped in discovery of the roles lncRNA in disease mechanisms which have been recorded in disease-association databases. For instance LNCipedia provides lncRNA from humans with experimental and putative annotations along with miRNA-lncRNAs associations (Volders et al., 2013). Similarly, lncRNAdb is repository for functionally annotated lncRNAs along with TF-lncRNA associations. LncRNome, a lncRNA database for human complied form GENCODE has lncRNAs with annotations of their biomolecular interactions and disease associations. LncATLAS provides information on lncRNA localization in cells from RNA-sequencing data, from GENCODE (Mas-Ponte et al., 2017), lnc2CAncer has 1,488 entries of lncRNAs from experimentally supported validations which are associated with cancer (Ning et al., 2016). Table 3 contains a list of databases and their references.

**Table 3:** Overview of databases of LncRNAs.

**Database**	**Description**	**References**
NONCODEV5	Knowledge base for ncRNAs	Fang et al., 2018
LNCipedia	lncRNA with secondary structure prediction, protein coding potential and microRNA binding sites	Volders et al., 2013
lncRNAdb v2.0	Manually curated lncRNAs from literature	Quek et al., 2015
LncATLAS	lncRNA annotated with subcellular localisatiom	Mas-Ponte et al., 2017
lncRNAdisease 2.0	Experimentally supported lncRNA disease association and molecular targets	Bao et al., 2019
LncRBase	lncRNA with information about their subtypes and interactions	Chakraborty et al., 2014
lncRNome	lncRNA with interactions with other RNAs	Bhartiya et al., 2013
GreeNC v1.1.2	Database for plant lncRNAs	Paytuvi Gallart et al., 2016
Lnc2Cancer v2.0	Manually curated database with experimentally supported lncRNA-cancer associations	Gao et al., 2019b
EVLncRNAs	Manually curated database with validated with low-throughput experiments	Zhou et al., 2018
ChIPBase v2.0	lncRNAs and other ncRNA from ChIP seq data	Zhou et al., 2017
DIANA-LncBase v3	Database dedicated to cataloging miRNA and lncRNA interactions	Karagkouni et al., 2020
LNCediting	Information of lncRNA editing, its impact and interactions with miRNAs	Gong et al., 2017
TCLA	Cancer LncRNome Atlas: lncRNAs predicted from TCGA datasets	Yan et al., 2015
MNDR v2.0	Experimental and predicted ncRNA-disease associations	Cui et al., 2018
lncRNASNP2	lncRNA variants and their disease associations	Miao et al., 2018
Lnc2Meth	Manually curated database of regulatory relationships between long non-coding RNAs and DNA methylation associated with human disease	Zhi et al., 2018
DES-ncRNA	Database of human miRNA and lncRNA from literature	Salhi et al., 2017
LincSNP2.0	disease associated SNPs with lncRNAs	Ning et al., 2017
LncVar	lncRNAs with associated genetic variations	Chen et al., 2017
deepBase v2.0	ncRNA database from deep sequencing data	Zheng et al., 2016
C-It-loci	Tissue specific transcriptome data (protein coding genes and ncRNA)	Weirick et al., 2015
LncRNA2Target v2.0	lncRNA and lncRNA-to-target genes after lncRNA knockdown and over expression	Cheng et al., 2019
LncTarD	Manually curated database of lncRNAs and target regulations	Zhao et al., 2020
CRlncRNA	Cancer related lncRNAs along with associations and interactions	Wang et al., 2018
lncRNAKB	Cancer related lncRNAs along with associations and interactions	Seifuddin et al., 2020
Cancer LncRNA Census (CLC)	lncRNAs from GENCODE involved in cancer	Carlevaro-Fita et al., 2020

## Case Study: Co-Expression Network Analysis Identifying Pro-Inflammatory lncRNAs Implicated in HCC

**Figure 2:**
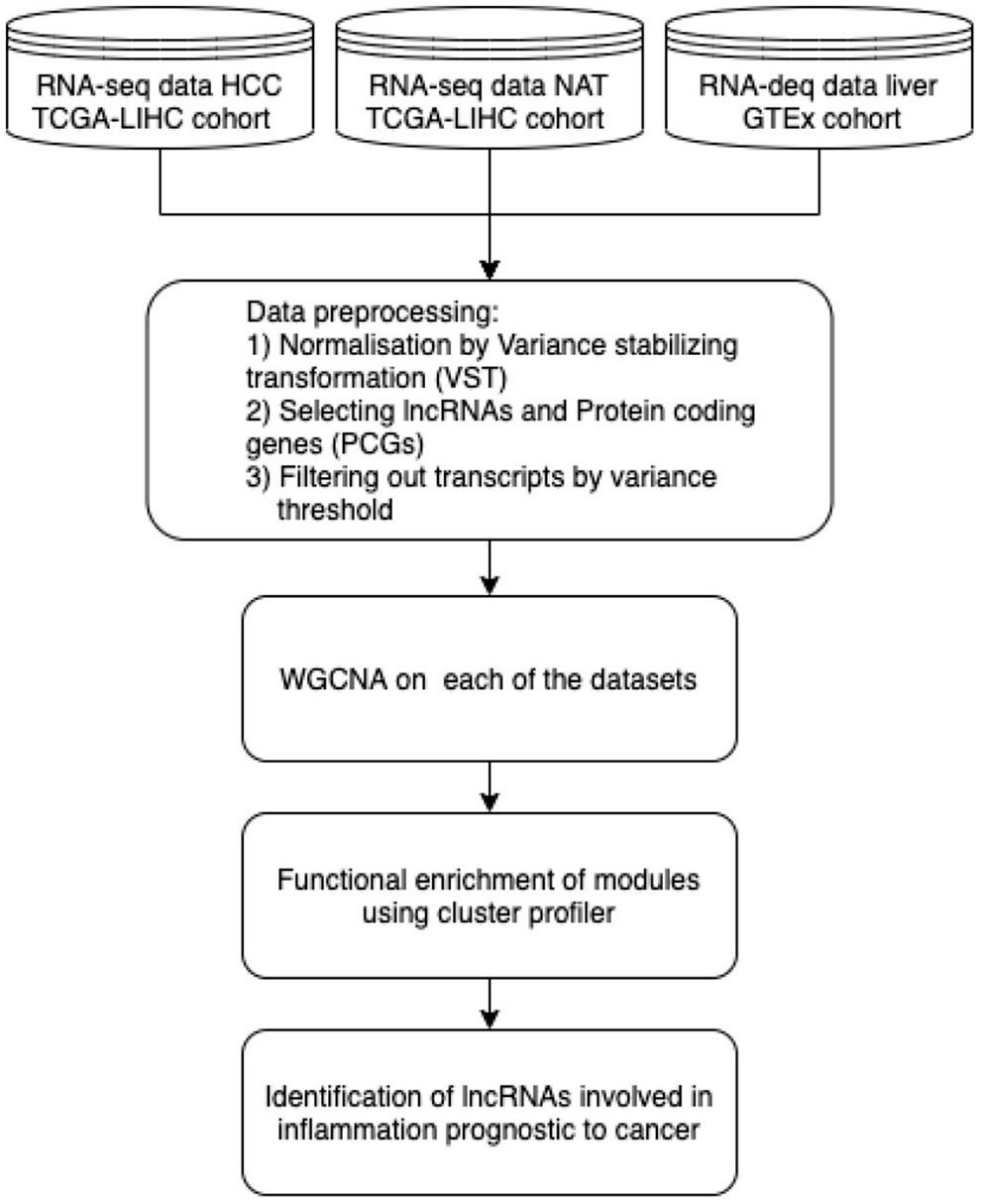
RNA-seq-based co-expression network analysis pipeline for identification of lncRNAs in pathways dysregulated in HCC from the TCGA-GTEx datasets.

Cancer is caused by continuous accumulation of unfavourable genetic alterations that cause deregulation of genetic networks and cellular pathways ultimately (Huarte, 2015) leading to unceasing growth of cells and tissue. The mechanisms of these dysregulations are complex, involving altered gene expressions and molecular interactions which are yet to be discovered comprehensively; thus leading to the necessity to analyze the anomalies at all omics levels. In fact, LncRNAs are diversely associated in most of the hall marks of cancer. Many of the studies on cancer associated lncRNAs have mainly analyzed expression profile variations of lncRNA in cancer vs. healthy tissue and its effects on deregulated pathways and identification their regulatory targets. Also, approaches to identify RNA folding and stable complexes to evaluate lncRNA functions have depicted that genetic alterations like SNPs can also majorly impact the RNA structure and eventually their function with changes in active/binding sites of lncRNAs (Wan et al., 2014; Schmitt and Chang, 2016). Chronic inflammation has known be a vital in cancer progression in case of Hepatocellular carcinoma(HCC). Some of the pathways known to be chronically upregulated causing hepatoma cell profileration include JAK/STAT signalling, NF-Kappa B signalling, PI3K/AKT/mTOR pathway, WNT pathway, and MAPK pathway (Chen et al., 2018; Yang et al., 2019). In order to investigate the application of co-expression network based on the “guilt by association” principle analysis of RNA-seq data, we applied the Weighted Gene Co-expression Network Analysis (WGCNA) (Langfelder and Horvath, 2008) on the following datasets: The RNA-Seq dataset from The Cancer Genome Atlas (Tomczak et al., 2015) Liver Hepatocellular Carcinoma (TCGA-LIHC) project and the GTEx dataset (Lonsdale et al., 2013) (Table 4) samples to identify the pathways dysregulated in HCC with regards to chronic inflammation in HCC progression. The steps in the pipeline are illustrated in Figure 2. The datasets were collected and analyzed using the TCGAbiolinks, WGCNA packages in R.

**Table 4:** Details of datasets used in the case study.

**Dataset**	**Project**	**Number of samples**	**Number of modules**	**Data source**
RNA-Seq expression data HCC	TCGA-LIHC	372	27	Tomczak et al., 2015
RNA-Seq expression data NAT	TCGA-LIHC	50	76	Tomczak et al., 2015
RNA-Seq expression from liver	GTEx	208	43	Lonsdale et al., 2013

WGCNA analysis consists of the following steps: correlations across the normalized expression values of the samples are computed and raised to a soft threshold power based on the scale free topology criterion generating an adjacency matrix representing the co-expression network. This is followed by hierarchical clustering is used to identify clusters of co-expressed lncRNAs and protein coding genes among the network, each of which is labeled with a color/number. Co-expression Network using WGCNA was generated across all the 3 datasets and modules obtained in each case were enriched for functional process by cluster profiler. The modules which were identified for pathways dysregulated in case of HCC were selected and the lncRNAs which were highly connected, i.e., being significant for each module were identified for having bio-marker prognostic potential.

**Figure 3:**
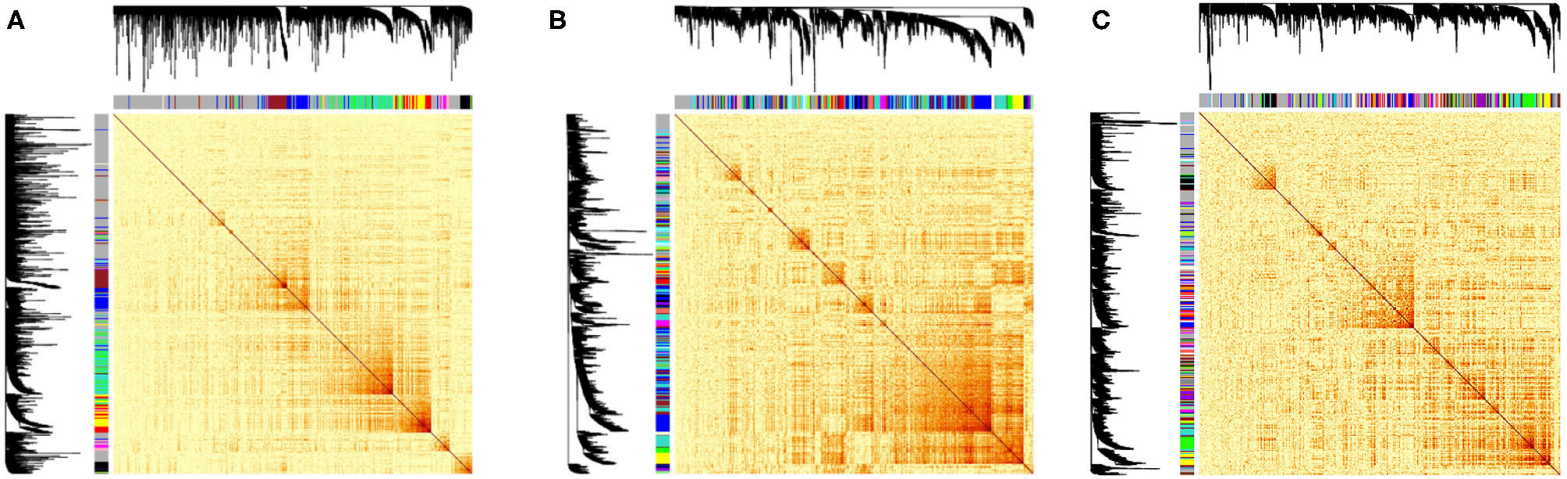
Heatmaps of the correlations between lncRNAs-mRNAs with their corresponding cluster dendrograms of the datasets. The colors below the dendrogram indicate the clusters. **(A)** NAT tissue TCGA-LIHC project, **(B)** HCC tissue TCGA-LIHC project, and **(C)** Liver tissues from GTEx project.

For HCC, NAT and GTEx profiles 27, 76 and 43 modules were identified, respectively from the hierarchical clustering with the cut height being selected 0.99, 0.98, 0.98 (Figure 3), respectively. These includes all the PCGs and lncRNAs transcripts. Each module was labeled with a color allocated by the WGCNA function and were enriched for KEGG pathways with threshold *p* < 0.05. The *red, yellow* in TCGA-HCC dataset and *turquoise, green* modules in TCGA-NAT dataset were enriched for the pathways involved in inflammation including JAK-STAT signaling pathway, cytokine-cytokine receptor interaction, NF-kappa B signaling pathway, T cell receptor signaling pathway among others contributing in inflammatory response. The network properties of all the networks were calculated based on which the transcripts in these modules were sorted according to their connectivity. The top highly connected lncRNAs(top 10) putatively having important regulatory mechanisms in these modules were selected for having biomarker potential in regards to chronic inflammation both in the tumour and its is surrounding micro environment proceeding to NAT. The common lncRNAs among the both phenotypes across these modules were *PCED1B-AS1, TRG-AS1, MIR155HG, MIAT, LINC00996*. MIAT has been known to be implicated in several cancers such as breast cancer, gastrointestinal cancer and NSCLC and also its silencing has known to inhibit cell proliferation and tumorogenesis in HCC (Zhao et al., 2019). In a recent study by Peng et al. (2020) it has been postulated that *MIAT* regulates the expression of JAK2 among other genes and has an important role in controlling the tumour microenvironment in HCC.

LINC00996 has also been known to have regulatory mechanism in the JAK-STAT signalling pathway in colorectal cancer in a study by Ge et al. (2018). These pathways are dysregulated in the case of HCC as seen in the clusters from the TCGA datasets (HCC and NAT) but not the GTEx dataset. This provides us with corroboration pointing that NAT is subjected to an inflammatory environment prompted by the malignant tissue. This is similar to micro tumour environment with higher proliferation rate than a healthy hepatocyte. Identification of these modules and lncRNAs provides extended empirical evidence of lncRNA regulation in inflammation and pertaining to cancer progression. This analysis provides support to the “guilt by association” hypothesis of co-expression of lncRNAs with the genes involving in similar functions. However, few of the lncRNAs like MEG3, MALAT1, H19, UCA1 which have been studied for their implications in HCC didn't show an expression in the GTEx greater the variance threshold and could not be characterized in the co-expression networks while comparing to the TCGA datasets. This could be attributed to the batch effects of the RNA-Seq experiments across the GTeX and TCGA projects which can be addressed and corrected while pre-processing the raw reads together from all the datasets. The understanding of such complex networks in which dysregulation of lncRNAs occurs impacting cancer progression and metastasis, which also being tissue specific can set lncRNAs to become excellent biomarkers in cancer therapy (Schmitt and Chang, 2016).

## Concluding Remarks

The recent discovery of the lncRNAs in the non-coding genome has led to a paradigm shift in the understanding of the mechanism of information flow from the genetic code and the genotype-phenotype map. But, as discussed, the mechanisms in which lncRNAs functions are very complex involving interactions with various molecules from other 'omic' levels. Advancements in RNA technologies have helped to elucidate some of the diverse mechanisms of lncRNAs but the regulatory potential of the majority of these noncoding genes have yet to be discovered. Differential co-expression of lncRNAs, RNA secondary structure and sequence analysis and prediction, ML based approaches in computational pipelines have aided in the identification and characterization of lncRNAs from RNA-seq experiments. This has to be supported by experimental validations and clarifications on cis-trans regulatory processes. Genome wide transcriptome profiling has identified several lncRNAs which have significant roles in diseases like cancer exhibiting cell- and/or tissue/tumor-specific expression and hence can be excellent candidate targets for therapy. It has been demonstrated that silencing of certain disease associated lncRNAs exhibited tumor suppression. In summary, a comprehensive knowledge of lncRNAs shall provide researchers insights into genotype-phenotype distinction and genetic disorders leading to more effective therapeutic strategies for diseases and with emergence of new experimental designs and computational pipelines we can advance our understanding of the transcriptome.

## Author Contributions

VS and RS: original idea for the manuscript, contributed to design and conceptualization of the study, and supervision. AC: literature, data analysis, and writing initial draft. VS: writing, review, and editing. RS: project administration and funding acquisition. All authors also critically reviewed, wrote, and approved the final version.

## Conflict of Interest

The authors declare that the research was conducted in the absence of any commercial or financial relationships that could be construed as a potential conflict of interest.
